# Draft Genome Sequence of *Ralstonia pickettii* AU12-08, Isolated from an Intravascular Catheter in Australia

**DOI:** 10.1128/genomeA.00027-14

**Published:** 2014-02-06

**Authors:** Li Zhang, Mark Morrison, Claire M. Rickard

**Affiliations:** a Centre for Health Practice Innovation, Griffith Health Institute, Griffith University, Brisbane, Australia; b Diamantina Institute, the University of Queensland, Brisbane, Australia

## Abstract

*Ralstonia pickettii* is a nonfermenting Gram-negative bacillus that creates a significant problem in clinical settings, as it is a widespread cause of nosocomial infections. Here, we report the draft genome sequence of *R. pickettii* AU12-08, isolated from an intravascular catheter tip.

## GENOME ANNOUNCEMENT

*Ralstonia pickettii* was previously known as *Pseudomonas pickettii* and *Burkholderia pickettii* (1). *R. pickettii* is an aerobic Gram-negative, oxidase-positive, nonfermenting rod that has been isolated from a wide variety of clinical specimens, including blood, urine, and cerebrospinal fluid (2). *R. pickettii* is not considered to be a major pathogen, and its virulence level is thought to be low (3). However, a wide range of *R. pickettii* infections have been reported recently (4). This demonstrates that this organism might be a more widespread pathogen than was thought. In addition, the types of infections are more invasive and severe than was thought (4).

*R. pickettii* AU12-08 was isolated from an intravascular catheter tip by rolling the tip back and forth on the surface of a Columbia agar plate supplemented with 5% sheep blood, essentially as described by Maki et al. (5). DNA was prepared and the genome sequence of *R. pickettii* AU12-08 was determined on a 454 GS FLX system using Titanium chemistry (Roche) (6). The sequence data consist of 135,359,388 bp of DNA sequence at 22× coverage. A total of 78 contigs (>500 bp) were *de novo* assembled using the Roche GS *de novo* assembler (version 2.3). The contig N_50_ is 178,545 bp, and the largest contig assembled is 592,110 bp. The contigs were then ordered and oriented into 14 scaffolds using paired-end information. The average length of the scaffolds is 446,804 bp.

The draft genome of *R. pickettii* AU12-08 consists of a circular 6,229,152-bp chromosome, with a G+C content of 63.6%. The genome was automatically annotated using the RAST server (7). The genome contains 50 tRNA genes coding for all amino acids and 5,733 predicted protein-coding genes, consistent with other sequenced *Ralstonia* spp. (8, 9). We identified numerous putative virulence factors, including those involved in quorum sensing and biofilm formation, as well as the production of bacteriocins and invasins. The *R. pickettii* AU12-08 genome contains 22 putative resistance-nodulation-cell division multidrug resistance efflux pumps, and 13 genes code for multidrug resistance. Four genes code for resistance to fluoroquinolones and 10 genes code for β-lactam antibiotics. In addition, 170 genes code for resistance to toxic compounds, including cobalt-zinc-cadmium resistance, copper homeostasis, mercury resistance, and arsenic and bile hydrolysis.

The sequence of the *R. pickettii* AU12-08 genome will greatly improve our understanding of the drug resistance and pathogenicity of this organism.

### Nucleotide sequence accession number.

The genome sequence of *R. rickettii* AU12-08 has been deposited in NCBI GenBank under the accession no. ASZV00000000.
